# Real-world efficacy and safety of insulin degludec with mealtime rapid-acting insulin in type 1 diabetes in Indian pediatric population

**DOI:** 10.1186/s13633-018-0059-0

**Published:** 2018-07-27

**Authors:** Inderpal Singh Kochar, Aashish Sethi

**Affiliations:** Indraprastha Apollo Hospital, New Delhi, India

**Keywords:** Adolescents, Children, Hypoglycemia, Insulin Degludec, Type 1 diabetes

## Abstract

**Background:**

Insulin Degludec (IDeg) is a new ultra-long-acting basal insulin that has not been yet evaluated in Indian pediatric population. We aim to evaluate the efficacy and safety of IDeg as basal-bolus therapy in Indian pediatric patients affected by type 1 diabetes mellitus (T1DM).

**Methods:**

A total of 30 pediatric and adolescent patients (17 boys, 13 girls; 22 were pre-pubertal) with T1DM who were on IDeg once daily participated in the study. All the patients received IDeg for at least 26 weeks along with rapid-acting mealtime insulin and their pre- and post-baseline characteristics (anthropometric data (BMI), age, duration of diabetes), metabolic (HbA1C), insulin requirement (unit/kg body weight per day) and number of hypoglycemia episodes were recorded along with the daily self-monitoring of blood glucose.

**Results:**

There was a significant decline in HbA1c, FPG and bolus insulin dose from baseline to 26 weeks in the overall population (HbA1c: 9.65 ± 1.998% to 8.60 ± 1.631%, *P* = 0.0014; FPG: 156.93 ± 42.373 mg/dL to 109.37 ± 28.531 mg/dL, *P* = 0.000004; bolus insulin dose: 0.49 ± 0.208 U/kg/day to 0.35 ± 0.155 U/kg/day, *P* = 0.00032). The basal insulin dose was significantly higher at 26 weeks compared to baseline dose (0.42 ± 0.134 U/kg/day to 0.46 ± 0.139 U/kg/day, *P* = 0.04219). There was no significant change in BMI at 26 weeks.

None of the patients experienced any DKA episode for 26 weeks. 16.7% patients had experienced at least one symptomatic hypoglycemia episode. On CGMS among the patients who were shifted from Glargine to degludec hypoglycemia were reduced significantly (overall hypoglycemia: 1.92 ± 1.26 to 0.35 ± 0.49 episodes over 3 days, *P* = 0.0026 while nocturnal hypoglycemia: 0.92 ± 0.47 to 0.21 ± 0.42 episodes, *P* = 0.0021). None of the patients had severe hypoglycemia episode.

**Conclusion:**

In our study IDeg is found to be safe and effective long-acting basal insulin that can be used in Indian pediatric population with T1DM. However further long term prospective studies are required to evaluate the long term effects.

## Background

Type 1 diabetes mellitus (T1DM), is one of the most common pediatric endocrine illnesses [1]. Globally, ~ 78,000 children under 15 years of age are estimated to develop T1DM annually. Recent reports have estimated that 24 and 23% of pediatric diabetic patients belong to European and South-East Asian regions, respectively [2]. India alone is a home of around 97,700 children with T1DM [1].

T1DM management in children and adolescents is a challenging task due to their pubertal or hormonal changes, variable daily schedules, flexible and irregular meal timings, fear of needles, correct injection-site rotation and/or subcutaneous injection depths, pain during injections, social stigma and varying family support [3]. The complex interaction between these trajectories has shown limited success of medical or psychological interventions so far [4]. Hypoglycemia, particularly nocturnal hypoglycemia, is considered as the major barrier in achieving glycemic control [5]. Long-term sequelae of hypoglycemic episodes may impair neuropsychological functioning and decrease quality of life [6]. This fear of hypoglycemia frequently results in insufficient insulin supplementation, which may lead to hyperglycemia and diabetic ketoacidosis (DKA) [7]. Various studies have shown that reduced cognitive ability was more closely related to the presence of microangiopathy, suggesting that chronic hyperglycemia has a greater adverse impact on the brain [8]. The Clinical Practice Consensus Guideline recommends optimal glycemic control without severe hypoglycemia to be the main target in T1DM management in children and adolescents [9].

Basal-bolus insulin therapy plays a pivotal role in lowering the glycemic level where long-acting basal insulin analogues have long duration of action, low nocturnal hypoglycemic risk and less variability. However, they only lasts for 24 h and start decreasing at the end hours, thereby leading to occasional hyperglycemia just before the next insulin dose & requiring next dose to be given at fixed time of the day [10]. Hence, insulin Degludec (IDeg), an ultra-long-acting basal analog approved by the US Food and Drug Administration (FDA) on September 25, 2015 was developed with the properties of having a flat peakless profile, less day-to-day variability, once-daily dosing, flexible timing of administration, long-acting profile (> 42 h), low overall and nocturnal hypoglycemia, a mean elimination half-life of ~ 25 h and an ability to mix with other insulins. On subcutaneous injection, it self-associates and forms a depot of soluble multihexamers that further allows a stable and slow release of monomers into the circulation [11].

A battery of literature supports the effective and safe use of IDeg over other basal insulin analogs in T1DM adult population in terms of significant glycemic control with a lower risk of hypoglycemia, nocturnal hypoglycemia, dose requirement and higher flexibility [12–14]. However, only few IDeg studies have been reported in children and adolescents with T1DM. The efficacy and safety of IDeg have not been evaluated in Indian pediatric population with T1DM. Hence, the present study was undertaken to assess the real-world efficacy and safety of IDeg in basal-bolus treatment, with mealtime rapid-acting insulin, in Indian pediatric T1DM patients.

## Methods

### Study design

This was a 26-week, non-randomized, open-label study involving retrospective data collection from 30 children and adolescents with T1DM. All the patients attended the pediatric outpatient department of the Indraprastha Apollo hospital, Delhi between February 2016 to February 2017. The study enrolled T1DM patients who were on treatment with Long acting Insulin degludec along with rapid acting mealtime insulin (Aspart/Lispro/Glulisine) for at least 26 weeks and maintained blood glucose diary with atleast 3 point SMBG daily, and have continuous glucose monitoring system (CGMS) data using Medtronic iPro2 Professional CGM (at time of switching from insulin Glargine to degludec). The study was initiated after taking an approval from institutional ethics committee and was conducted in accordance with principals of the Declaration of Helsinki and ICH Good Clinical Practice.

The anthropometric data [body mass index, height, weight, Standard Deviation Score(SDS) as per standard growth charts (< 5 years-WHO, 5–18 years-IAP 2015 Growth curves)], age, HbA1c, basal and bolus insulin requirement (unit/kg bodyweight/day), SMBG data from diabetic diary (Fasting sugar taken as average of 7 days fasting glucose reading) and the safety variables were recorded into the paper case record form at baseline and 26 weeks’ post-baseline. The safety variables included episodes of hypoglycemia [self-measured plasma glucose level < 70 mg/dL, regardless of symptoms, by collecting data from self-maintained Blood Glucose diary, hypoglycemia recorded on CGMS iPro2 (for 3 days at shifting from Glargine to degludec and after 26 weeks)], hyperglycemia (prandial glucose levels > 200 mg/dL) and DKA, lipodystrophy, local site reactions and any other adverse events (AEs). The data entry was further validated and analyzed statistically.

### Study endpoints

The primary endpoint of the study was change in HbA1c level from baseline to 26 weeks. The secondary endpoints were changes in BMI, insulin dose (bolus, basal and total) and mean FPG levels from baseline to 26 weeks. The safety parameters were also evaluated during the study. The exploratory endpoint was to assess the changes in HbA1c, BMI, insulin dose and FPG from baseline to 26 weeks by gender, age (1–6 years, 6–12 years and > 12 years) and comparison from previous basal insulin.

### Statistical analyses

The descriptive statistics for continuous variables like HbA1c, BMI, FPG was presented with number (N) of non-missing observations, mean, standard deviation, median, minimum and maximum (range). For categorical data, descriptive statistics was presented with a number (N or n) and their percentages. All the statistical tests were done at 5% level of significance.

Changes from baseline to week 26 for HbA1c, BMI, FPG and insulin dose were analyzed using paired t-test to test if there was a significant difference between baseline and week 26. Paired t-test was also applied individually on 3 age groups and gender. The t-test was used to test significant difference between age groups and males and females.

## Results

Out of 189 Type 1 Diabetes patients who were on basal-bolus insulin therapy with insulin degludec, 30 patients matched the inclusion criteria, 17 were males and 13 were females. 20 patients (Duration of diabetes 3.09 ± 2.61 years) were shifted from Glargine to insulin degludec (Group A) (Basal insulin decreased by 10% while bolus insulin kept as same) and 10 patients were insulin naïve patients (Group B) in whom basal characteristics (BMI, HbA1c, FPG, basal and bolus insulin doses) were retrospectively collected at 1 month after diagnosis. Baseline characteristics of the overall population and by stratified age groups are summarized in Table 1. The comparison of BMI, HbA1c, FPG, basal and bolus insulin doses at baseline and 26 weeks post IDeg treatment in the overall population, both and by age groups (1–6 years, 6–12 years and > 12 years), gender & in both groups is shown in Table 2, Table 3 and Table 4 respectively. The comparison of hypoglycemic episodes recorded in CGMS shown in Table 5.

**Table 1:** Baseline Characteristics

Characteristics	Total Population	Age categories		
		1–6 years	6–12 years	> 12 years
Number of Patients	30	8	14	8
Female/Male	13/17	2/6	6/8	5/3
Age (years)	9.23 ± 3.942	4.54 ± 0.962	9.07 ± 1.619	14.22 ± 2.229
Weight (kg)	30.11 ± 13.161	18.11 ± 4.209	29.10 ± 10.627	43.88 ± 10.346
Weight (SDS)	− 0.15 ± 1.41	0.27 ± 1.35	− 0.06 ± 1.00	− 0.59 ± 0.78
Height (cm)	130.09 ± 18.906	108.19 ± 9.494	130.14 ± 11.374	151.89 ± 7.691
Height (SDS)	−0.04 ± 1.13	0.39 ± 1.56	−0.27 ± 1.39	−0.54 ± 1.15
BMI (kg/m^2^)	16.99 ± 3.364	15.28 ± 1.274	16.79 ± 3.297	19.04 ± 4.108
BMI (SDS)	0.04 ± 0.97	0.04 ± 0.81	0.10 ± 0.91	−0.43 ± 0.89
HbA1c (%)	9.65 ± 1.998	8.59 ± 0.753	10.32 ± 2.213	9.53 ± 2.159
FPG (mg/dL)	156.93 ± 42.373	136.50 ± 12.862	176.43 ± 49.781	143.25 ± 34.919
Insulin dose (U/kg of body weight/day)				
Basal insulin	0.42 ± 0.134	0.38 ± 0.120	0.46 ± 0.165	0.40 ± 0.069
Bolus dose	0.49 ± 0.208	0.37 ± 0.115	0.57 ± 0.255	0.48 ± 0.128

*BMI* body mass index, *FPG* fasting plasma glucose, *HbA1c* glycated hemoglobin, *SDS* Standard Deviation Score

**Table 2:** Comparison of BMI, HbA1c, FPG, Basal and Bolus Insulin Doses at Baseline and 26 weeks after IDeg Treatment by Age

Characteristics	Baseline	26 weeks
BMI (kg/m^2^) (SDS)		
Overall population (*N* = 30)	16.99 ± 3.364 (0.04 ± 0.97)	17.39 ± 3.005 (0.15 ± 0.89)
1–6 years (*n* = 8)	15.28 ± 1.274 (0.04 ± 0.81)	15.83 ± 1.199 (0.48 ± 0.63)
6–12 years (*n* = 14)	16.79 ± 3.297 (0.10 ± 0.91)	17.18 ± 2.653 (0.20 ± 0.91)
> 12 years (n = 8)	19.04 ± 4.108 (− 0.16 ± 1.11)	19.33 ± 3.964 (− 0.15 ± 1.06)
HbA1c (%)		
Overall population (N = 30)	9.65 ± 1.998	8.60 ± 1.631^*^
1–6 years (n = 8)	8.59 ± 0.753	8.31 ± 0.930
6–12 years (n = 14)	10.32 ± 2.213	8.50 ± 1.024^*#^
> 12 years (n = 8)	9.53 ± 2.159	9.06 ± 2.802
FPG (mg/dL)		
Overall population (N = 30)	156.93 ± 42.373	109.37 ± 28.531^*^
1–6 years (n = 8)	136.50 ± 12.862	105.75 ± 26.114^*^
6–12 years (n = 14)	176.43 ± 49.781	108.36 ± 28.678^*^
> 12 years (n = 8)	143.25 ± 34.919	114.75 ± 33.363^*^
Insulin dose (U/kg of body weight/day)		
Basal insulin		
Overall population (*N* = 30)	0.42 ± 0.134	0.46 ± 0.139^*^
1–6 years (n = 8)	0.38 ± 0.120	0.41 ± 0.109
6–12 years (n = 14)	0.46 ± 0.165	0.52 ± 0.163
> 12 years (n = 8)	0.40 ± 0.069	0.41 ± 0.074
Bolus insulin		
Overall population (N = 30)	0.49 ± 0.208	0.35 ± 0.155^*^
1–6 years (n = 8)	0.37 ± 0.115	0.27 ± 0.077^*^
6–12 years (n = 14)	0.57 ± 0.255	0.39 ± 0.176^*^
> 12 years (n = 8)	0.48 ± 0.128	0.37 ± 0.156^*^

*BMI* body mass index, *FPG* fasting plasma glucose, *HbA1c* glycated hemoglobin, *SDS* Standard Deviation Score

*P < 0.05 versus baseline (paired t-test)

^#^*P* < 0.05 versus 1–6 years (t-test)

**Table 3:** Comparison of BMI, HbA1c, FPG, Basal and Bolus Insulin Doses at Baseline and 26 weeks after IDeg Treatment by Gender

Characteristics	Baseline	26 weeks
BMI (kg/m^2^) (SDS)		
Female (*n* = 13)	17.73 ± 3.897 (0.07 ± 0.97)	18.07 ± 3.882(0.20 ± 0.82)
Male (*n* = 17)	16.42 ± 2.885 (− 0.02 ± 0.93)	16.87 ± 2.099(0.16 ± 0.88)
HbA1c (%)		
Female (*n* = 13)	9.35 ± 1.824	8.37 ± 0.772^*^
Male (n = 17)	9.88 ± 2.147	8.78 ± 2.074^*^
FPG (mg/dL)		
Female (n = 13)	157.77 ± 54.259	108.85 ± 25.980^*^
Male (n = 17)	156.29 ± 32.330	109.76 ± 31.126^*^
Insulin dose (U/kg of body weight/day)		
Basal insulin		
Female (n = 13)	0.45 ± 0.115	0.49 ± 0.136
Male (n = 17)	0.41 ± 0.148	0.44 ± 0.141^*^
Bolus insulin		
Female (n = 13)	0.57 ± 0.212	0.43 ± 0.161^*^
Male (n = 17)	0.44 ± 0.193	0.29 ± 0.122^*^

*BMI* body mass index, *FPG* fasting plasma glucose, *HbA1c* glycated hemoglobin, *SDS* Standard Deviation Score

*P < 0.05 versus baseline (paired t-test)

**Table 4:** Comparison of BMI, HbA1c, FPG, Basal and Bolus Insulin Doses at Baseline and 26 weeks after IDeg Treatment among patients who were previously on insulin Glargine (Group A) vs. previously insulin naïve (Group B)

Characteristics	Baseline	26 weeks
BMI (SDS)		
Group A (*n* = 20)	−0.11 ± 0.76	0.04 ± 0.73
Group B (n = 10)	0.38 ± 1.31	0.37 ± 1.19
HbA1c (%)		
Group A (n = 20)	8.50 ± 0.65	8.16 ± 0.67*
Group B (*n* = 10)	11.94 ± 1.78	9.47 ± 2.52*
FPG (mg/dL)		
Group A (n = 20)	135.80 ± 19.05	104.50 ± 20.69*
Group B (n = 10)	199.20 ± 45.18	119.10 ± 39.50*
Insulin dose (U/kg of body weight/day)		
Basal insulin		
Group A (n = 20)	0.45 ± 0.15	0.47 ± 0.14
Group B (n = 10)	0.36 ± 0.05	0.43 ± 0.13
Bolus insulin		
Group A (n = 20)	0.46 ± 0.20	0.32 ± 0.10*
Group B (n = 10)	0.54 ± 0.22	0.41 ± 0.21
Total insulin		
Group A (n = 20)	0.91 ± 0.33	0.79 ± 0.21*
Group B (n = 10)	0.91 ± 0.24	0.84 ± 0.31

BMI = body mass index; FPG = fasting plasma glucose; HbA1c = glycated hemoglobin, SDS = Standard Deviation Score

*P < 0.05 versus baseline (paired t-test)

**Table 5:** Comparisons of the frequencies of overall and nocturnal hypoglycemia (glucose< 70 mg/dl) events recorded on CGMS over 3 days when using Insulin Glargine and Insulin Degludec

		0 weeks	26 weeks	P
1	Total Hypoglycemia	1.92 ± 1.26	0.35 ± 0.49	0.0026*
2	Nocturnal Hypoglycemia	0.92 ± 0.47	0.21 ± 0.42	0.0021*

*P < 0.05 versus baseline (paired t-test)

### Comparison of BMI at 26 weeks after treatment

There was no significant change in BMI SDS from baseline to 26 weeks in the overall population and no changes in both groups. Similar results were reported when BMI data was compared by age groups. In addition, no significant difference in BMI at 26 weeks from baseline was reported between patients of different age groups.

### Comparison of glycemic levels (HbA1c and FPG) at 26 weeks after treatment

There was a significant decline in HbA1c and FPG levels from baseline to 26 weeks in the overall population (HbA1c: 9.65 ± 1.998% to 8.60 ± 1.631%, *P* = 0.0014; FPG: 156.93 ± 42.373 mg/dL to 109.37 ± 28.531 mg/dL, *P* = 0.000004).

There was a significant decline in HbA1c and FPG levels from baseline to 26 weeks in both groups (Group A HbA1C difference: 0.33 ± 0.52, *P* = 0.010 & FPG difference: 31.30 ± 22.21, *P* < 0.001 while Group B HbA1C difference: 2.47 ± 2.13, *P* = 0.005 & FPG difference: 80.10 ± 62.58, *P* = 0.003).

The HbA1c levels declined at 26 weeks from baseline in all the 3 age groups. However, this decline was only significant in the age group 6–12 years (10.32 ± 2.213% to 8.50 ± 1.024%, *P* = 0.0035). Between different age groups, the HbA1c decline was significantly higher in 6–12 year category compared to 1–6 year category (HbA1c difference: − 1.82 ± 1.918% versus − 0.28 ± 0.417%, *P* = 0.0375). There was no significant difference between other age categories.

Regarding FPG levels, there was a significant decline from baseline to 26 weeks in all the 3 age categories but no significant difference in the FPG level was reported between the age groups.

### Comparison of basal and bolus insulin doses at 26 weeks after treatment

The basal insulin dose (unit of insulin/kg of body weight/day) was significantly higher and bolus insulin dose was significantly lower at 26 weeks compared to doses at baseline in the overall population (basal insulin dose: 0.42 ± 0.134 U/kg/day to 0.46 ± 0.139 U/kg/day, *P* = 0.04219; bolus insulin dose: 0.49 ± 0.208 U/kg/day to 0.35 ± 0.155 U/kg/day, *P* = 0.00032).

There was increase in Basal insulin in both groups (A&B) (Group A Basal insulin difference: 0.02 ± 0.08 U/kg/day & Group B Basal insulin difference: 0.06 ± 0.11 U/kg/day) while it was not statistically significant. A decrease in bolus insulin in both groups (A&B) (Group A bolus insulin difference 0.14 ± 0.13 U/kg/day, *P* < 0.001, Group B bolus insulin 0.13 ± 0.27 U/kg/day, *P* = 0.160), and it was significant in patients who were previously on insulin Glargine (Group A).

There was an increase in basal insulin dose from baseline to 26 weeks in all the 3 age groups, but this change was not significant in either of the age groups. A significant decrease in bolus insulin dose at 26 weeks from baseline was reported across all the age groups. The difference in basal and bolus insulin doses at 26 weeks from baseline was not significant between the age groups.

### Comparison of BMI, HbA1c, FPG, basal and bolus insulin doses at 26 weeks after treatment within and between males and females

There was a significant decline in HbA1c and FPG levels and bolus insulin dose from baseline to 26 weeks in both genders. The basal insulin dose increased from baseline to 26 weeks in both genders, but the increase was significant only in males. No significant change in BMI from baseline to 26 weeks was reported in both genders (Table 3).

There was no significant difference in average BMI, HbA1c, FPG, basal and bolus insulin doses at 26 weeks post IDeg treatment between males and females.

### Safety parameters

Five (16.7%) and 13 (43.3%) patients had experienced at least one symptomatic hypoglycemia and hyperglycemic episodes, respectively. One (3.33%) patient had lipodystrophy who was on insulin treatment for past 5 years and 3 (10%) patients had local site reactions. No other AEs were reported in the study. None of the patients experienced any severe hyperglycemia episodes, and DKA episodes after IDeg treatment.

Nocturnal hypoglycemia was only observed during the continuous glucose monitoring system (CGMS) using Medtronic iPro2 Professional CGM among the patients who were previously on glargine, which was done for 3 days period just before starting of IDeg and after 26 weeks (Table 5); which showed significant reduction in nocturnal hypoglycemia (overall hypoglycemia: 1.92 ± 1.26 to 0.35 ± 0.49 episodes over 3 days, *p* = 0.0026 while nocturnal hypoglycemia: 0.92 ± 0.47 to 0.21 ± 0.42, *p* = 0.0021).

## Discussion

The present observational study aims to evaluate efficacy and safety of IDeg, a long-acting basal insulin, along with mealtime rapid-acting insulin for 26 weeks in children and adolescents with T1DM during their routine clinical care. During the study, T1DM patients showed a significant decline in their glycemic levels and daily bolus insulin doses at 26 weeks.

Severe and recurrent episodes of hypoglycemia in young children and adolescents may result in poor neurocognitive outcomes [8]. Hence, avoidance of hypoglycemic episodes in this age group should be given a major consideration. To maintain optimal glycemic control and a target HbA1c of < 7.5%, as suggested by The International Society for Pediatrics and Adolescent Diabetes (ISPAD), without associated hypoglycemic episodes is considered as one of the biggest challenges in children and adolescents. In our study, there was a significant increase in basal insulin dose/kg of body weight/day requirement at week 26 than at baseline, however this was not significant individually in the groups. However, another study evaluating efficacy and safety of IDeg in children and adolescents with T1DM reported a significant reduction in basal insulin dose from baseline to 6 months (21.8 ± 8.9 IU/day vs. 19.4 ± 7.8 IU/day, *P* = 0.003) [15]. Thalange and co-workers have also observed reduction in the mean daily basal insulin dose in IDeg group compared to IDet group at week 52 (0.38 U/kg versus 0.55 U/kg), though the difference did not reach statistical significance [16]. Similar results were observed when the daily IDeg dose at week 24 was compared to the baseline dose [17].

In our study, we report a significant decrease in bolus insulin dose from baseline to week 26. Similar results have been reported in the previous study, indicating a significant reduction in bolus insulin dose from baseline to 6 months (0.56 ± 0.13 to 0.50 ± 0.15 U/kg/day; *P* = 0.02) [18]. However, no significant changes in bolus insulin dose from baseline was reported in other studies [16, 17].

Thalange et al. reported equivalent HbA1c level with significant reduction in FPG with IDeg when compared to IDet [16]. In another study, there was no significant improvement in HbA1c but a significant decline in FPG was evident after 6 months of IDeg treatment [15]. The present study depicts significant reduction in HbA1C and FPG in overall population and among groups containing patients who were shifted from Glargine and insulin naïve patients.

The ISPAD recommends HbA1c < 7.5% as the target glycemic level in children and adolescents with T1DM, regardless of age [7]. Basal bolus regimens, with IDeg as a basal insulin, helps in achieving optimal glycemic controls with minimal or no episodes of hypoglycemia. Various studies have reported low frequency of nocturnal hypoglycemia and non-inferiority of IDeg with IGlar in terms of glycemic levels (HbA1c and FPG) in adult T1DM patients [16, 17]. This reduction in hypoglycemia with IDeg is probably due to lower day-to-day and within-day variability and stable glucose lowering effect, which might facilitate titration and enable strict glycemic control [19]. In our study, significant reduction in hypoglycemia (overall and nocturnal) was observed on CGMS among patients shifted from Glargine to degludec.

Besides being effective, IDeg was found to be safe in Indian pediatric population aged < 18 years. Only 5 of 30 patients had symptomatic hypoglycemia and there was significantly decreased number of nocturnal hypoglycemia. Our results were in parallel to the reported literature where IDeg was associated with numerically or significantly lower rates of nocturnal hypoglycemia in children and adolescents with T1DM [16, 17]. The reduced association of IDeg with nocturnal hypoglycemia, irrespective of patient’s age, was due to its lower pharmacodynamic variability during nighttime [18]. No episode of DKA was reported during the 26 weeks of the study while if look at the data among the patients who were previously on insulin Glargine; no episodes of DKA was found in past 6 months while patient were on glargine.

The study has few limitations. First, it was a single-arm and a non-controlled observational study, involving retrospective data collection. Second, the sample size was small for evaluating efficacy and safety of IDeg in children and adolescents. Third, it was a single center study, imposing centre-based biased results. Fourth, it was a short duration study, which limited viability of the data.

## Conclusion

IDeg treatment in Indian children and adolescents with T1DM results in significant glycemic control with reduced hypoglycemic episodes and bolus insulin doses over the period of 26 weeks. Reduction in nocturnal hypoglycemic episodes has been reported in the study. However further long term prospective studies are required to evaluate the long-term effects.
